# Analysis of factors influencing latent tuberculosis infection screening using QuantiFERON-TB Gold In-Tube in people living with HIV/AIDS

**DOI:** 10.3389/fcimb.2025.1738028

**Published:** 2026-01-19

**Authors:** Yiming Xu, Jincheng Li, Song Gao, Yuying Huang, Qi Zhang, Huan Ding, Shipeng Zhang, Shiya Shen, Zhuping Xu

**Affiliations:** 1 School of Public Health, Nanjing Medical University, Nanjing, China; 2 Department of Chronic Infectious Disease Control and Prevention, Yangzhou Center for Disease Control and Prevention, Yangzhou, China; 3 Department of Chronic Infectious Disease Control and Prevention, The Affiliated Wuxi Center for Disease Control and Prevention of Nanjing Medical University, Wuxi Center for Disease Control and Prevention, Wuxi, China

**Keywords:** HIV, latent tuberculosis infection, QFT, restricted cubic splines (RCS), tuberculosis

## Abstract

**Objective:**

To investigate the influencing factors associated with the risk of latent tuberculosis infection (LTBI) among people living with HIV/AIDS (PLWHA).

**Methods:**

A cross-sectional study was conducted from July 2023 to July 2024, recruiting PLWHA from Wuxi City and Yangzhou City, Jiangsu Province, China. Data were collected through on-site questionnaire surveys and review of patient disease management records. QuantiFERON-TB Gold In-Tube (QFT) was used to detect LTBI. Multivariate logistic regression analysis was performed to identify factors associated with LTBI among PLWHA. Restricted cubic splines (RCS) models were employed to analyze the dose-response relationships between CD4^+^ cell count, CD8^+^ cell count, and CD4/CD8 ratio with LTBI risk.

**Results:**

A total of 1184 PLWHA were enrolled, with 8.4% having concomitant LTBI. Multivariate logistic regression revealed that age group 45-<60 years (OR = 2.158, 95% *CI*: 1.339-3.478, *P* = 0.002) and CD4/CD8 ratio ≥1 (OR = 3.562, 95% *CI*: 1.627-7.800, *P* = 0.001) were independent factors associated with LTBI. RCS model fitting results demonstrated a gradually increasing nonlinear dose-response relationship between continuous changes in CD4/CD8 ratio and LTBI. The dose-response relationship between CD4^+^ cell count and LTBI risk exhibited an “initial increase followed by a decrease trend. The dose-response relationship between CD8^+^ cell count and LTBI risk showed a gradual declining trend.

**Conclusion:**

This study identified that middle-aged PLWHA (45-<60 years) might represent a subgroup with relatively higher LTBI prevalence, indicating that screening in this age group may hold greater potential value. Additionally, the CD4/CD8 ratio, in conjunction with QFT findings, could serve as a supplementary reference for LTBI risk assessment. These observations support a more tailored approach to LTBI screening in PLWHA, though their implementation should be validated in prospective studies.

## Introduction

Tuberculosis (TB) is a chronic infectious disease caused by *Mycobacterium tuberculosis* infection, primarily affecting the lungs (WHO, 2024). According to the Global Tuberculosis Report 2024 of the World Health Organization (WHO), approximately 10.8 million people worldwide developed TB in 2023, with about 1.25 million deaths, including 1.09 million HIV-negative individuals and 161000 people living with HIV/AIDS (PLWHA) (Schito et al., 2015; WHO, 2024). TB remains among the leading causes of infectious disease mortality globally, imposing a substantial disease burden (Schito et al., 2015; GBD 2019 Diseases and Injuries Collaborators, 2020). Within individual hosts, HIV/TB co-infection creates a bidirectional pathogenic synergy, accelerating immune deterioration and disease progression (Bruchfeld et al., 2015; Liebenberg et al., 2021). Following infection with *Mycobacterium tuberculosis*, the majority of individuals remain in a state of latent tuberculosis infection (LTBI), characterized by persistent immune responses to mycobacterial antigens without clinical manifestations of active TB. Approximately 5%-10% of LTBI cases in the general population progress to active TB (Jiang et al., 2022), whereas for PLWHA, the risk of LTBI progressing to active TB is 20–37 times higher than in HIV-negative individuals (Jiang et al., 2022).

CD4^+^ T lymphocytes are the primary target cells of HIV infection. HIV infection leads to progressive depletion of CD4^+^ T lymphocytes (Vidya Vijayan et al., 2017). Through effective antiretroviral therapy (ART), CD4^+^ cell count can gradually recover to near-normal levels (Xiao et al., 2022). However, despite effective viral suppression and CD4^+^ cell count recovery, immune activation often persists, and CD8^+^ cell levels remain difficult to normalize after ART (Helleberg et al., 2015). Therefore, relying solely on CD4^+^ cell count does not fully reflect the immune function recovery status of PLWHA.

The CD4/CD8 ratio is an important indicator for predicting immune dysfunction and disease progression in PLWHA. HIV infection typically causes CD4/CD8 ratio inversion, and while ART may restore the CD4/CD8 ratio, it rarely exceeds 1, especially in patients with delayed treatment initiation (Serrano-Villar et al., 2014). Even when CD4^+^ cell count exceed 500 cells/μL and viral load falls below the detection limit in PLWHA, CD4/CD8 ratio inversion persists for extended periods (Xiao et al., 2025). Compared with CD4^+^ cell count alone, the CD4/CD8 ratio is a more accurate indicator for evaluating immune status during ART.

This study employed QuantiFERON-TB Gold In-Tube (QFT) to detect LTBI among PLWHA in regions with low prevalence of TB, and explored factors associated with LTBI in the PLWHA. PLWHA with active TB, previous TB history, or indeterminate QFT results were excluded based on clinical manifestations, comprehensive physical examinations, imaging, and etiological test results.

## Materials and methods

### Study participants and design

This cross-sectional survey recruited PLWHA from Wuxi Fifth People’s Hospital and Yangzhou Third People’s Hospital, Jiangsu Province, China, between July 2023 and July 2024. QFT was performed on participants by the Yangzhou Center for Disease Control and Prevention and the Wuxi Center for Disease Control and Prevention. All participants provided written informed consent. Based on clinical manifestations, comprehensive physical examinations, imaging, and etiological test results, PLWHA with active TB and previous TB history were excluded. Inclusion criteria: 1. PLWHA with no prior history of tuberculosis. 2. PLWHA who signed the informed consent form. Exclusion criteria: PLWHA individuals were excluded if they met any of the following criteria: 1. Presence of cough and/or sputum production lasting 2 weeks or longer, with or without hemoptysis or blood-streaked sputum, accompanied by symptoms such as night sweats, fatigue, or intermittent/persistent low-grade fever in the afternoon. 2. Positive acid-fast bacilli (AFB) smear or positive *Mycobacterium tuberculosis* culture from sputum specimens. 3. Chest X-ray findings suggestive of active tuberculosis, including multiple nodular lesions, patchy or fluffy infiltrates, lobar consolidation, mass-like opacities, and/or hilar or mediastinal lymphadenopathy.

### Procedures

PLWHA who presented to Wuxi Fifth People’s Hospital and Yangzhou Third People’s Hospital were encouraged to participate in the study after active tuberculosis had been excluded through medical history inquiry, chest X-ray examination, and sputum testing, including acid-fast bacilli (AFB) smear and/or *Mycobacterium tuberculosis* culture, prior to undergoing the QFT test. For eligible participants, trained investigators conducted questionnaire surveys to collect socio-demographic characteristics (including gender, age, ethnicity, occupation, residence, education level), body mass index (BMI), smoking and alcohol consumption history, underlying diseases, history of contact with TB patients, and TB history. Venous blood anticoagulated with lithium heparin was collected from all participants for QFT testing. Venous blood anticoagulated with ethylenediaminetetraacetic acid (EDTA) was collected for HIV viral load and CD4^+^ and CD8^+^ T lymphocyte measurements. In the absence of a universally accepted gold standard for latent tuberculosis infection, LTBI in this study was operationally defined as a positive QFT result in participants without clinical, radiological, or microbiological evidence of active TB, without a history of previous TB, and without indeterminate QFT results.

### Laboratory detection

The laboratory tests were conducted following the manufacturer’s instructions. The QuantiFERON-TB Gold In-Tube detection kit (Qiagen, Germany) was used for LTBI detection, and the result of QFT was determined using a QFT-GIT analysis software provided by the manufacturer (QIAGEN, 2016). HIV viral load was quantified using the COBAS AmpliPrep system (Roche Molecular Systems, 2007). The BD FACS Count™ system (BD Biosciences, USA) was employed for the detection of CD4^+^ and CD8^+^ T lymphocytes (BD Biosciences, 2015).

### Data grouping

PLWHA were divided into three age groups: young adults (<45 years), middle-aged (45-<60 years), and elderly (≥60 years); because there were only three participants younger than 18 years and relatively few participants aged ≥75 years, the former were included in the <45 year group and the latter were combined with the 60–74 year group in the ≥60 year category. Based on the Chinese Guidelines for Diagnosis and Treatment of HIV/AIDS (2024 Edition) (Acquired Immunodeficiency Syndrome Professional Group et al., 2024), CD4^+^ cell count was categorized into two groups: AIDS stage: <200 cells/μL; non-AIDS stage: ≥200 cells/μL. According to WHO classification criteria (WHO, 2021), HIV viral load was divided into three groups: viral suppression group: <50 copies/mL, low viral load group: 50-<1000 copies/mL, high viral load group: ≥1000 copies/mL. Based on a systematic review of CD4/CD8 count ratios (Ron et al., 2024), which identified a threshold of 0.3 to distinguish the risk of non-AIDS events in PLWHA, and combined with the Chinese Guidelines for Diagnosis and Treatment of HIV/AIDS (2024 Edition), the CD4/CD8 ratio was divided into three groups: <0.3, 0.3-<1, and ≥1.

### Statistical analysis

Normally distributed quantitative data were expressed as mean and standard deviation (Mean ± SD), and comparisons between groups were performed using independent samples t-test. Non-normally distributed quantitative data were expressed as median and interquartile range M (*P*25, *P*75). Qualitative data were described using frequency and proportion. An initial comparison of group differences was performed using univariate logistic regression analysis. The inclusion of variables in the multivariate logistic regression model was guided by a directed acyclic graph (DAG), which was constructed based on causal knowledge derived from previous studies. This approach allowed for the identification of true confounders in the exposure-outcome relationship, ensuring that only those covariates that were true confounders were adjusted for in the multivariate analysis. Odds ratios (OR) and 95% confidence intervals (*CI*) were calculated, with the significance level set at α=0.05 (two-sided). Restricted cubic splines (RCS) model was employed to explore the dose-response relationship between continuous changes in CD4/CD8 count ratio and LTBI risk. With LTBI as the outcome indicator and CD4/CD8 ratio as the independent variable, an RCS model was established to fit the dose-response relationship between CD4/CD8 ratio and LTBI risk in PLWHA. A restricted cubic spline model with 4 knots was selected based on the Akaike information criterion (AIC). The significance level was set at α=0.05. Excel was used to organize the raw questionnaire data, and R 4.3.3 software was used for data analysis.

## Results

### General characteristics

A total of 1184 eligible PLWHA were included, with ages ranging from 13 to 89 years and a median age of 45 years (IQR: 36–58 years). The population was predominantly male (90.1%, 1067/1184) and Han Chinese (98.6%, 1167/1184). The majority had not completed college or university education (64.3%, 761/1184), most resided in urban areas (70.4%, 834/1184), and enterprise employees represented the largest occupational group (40.0%, 474/1184). Among the study participants, 82.7% (979/1184) had no history of TB contact, 60.5% (716/1184) had a BMI between 18.5-24, 64.8% (767/1184) were non-smokers, and 69.7% (825/1184) were non-drinkers. Regarding underlying diseases, 80.2% (949/1184) of PLWHA had no comorbidities. Among PLWHA with other underlying diseases, cardiovascular disease was most common, accounting for 12.2% (144/1184) of enrolled subjects (**Table 1**).

**Table 1:** Comparison of baseline characteristics between the two groups.

Variable	Non-LTBI	LTBI	Total N (%)	Positive Rate	OR (95%CI)	*P* value
Gender						
Male	977 (90.1%)	90 (90.0%)	1067 (90.1%)	8.4%	1.000 (Ref)	–
Female	107 (9.9%)	10 (10.0%)	117 (9.9%)	8.5%	1.015 (0.512, 2.009)	0.967
Age						
<45	538 (49.6%)	38 (38.0%)	576 (48.6%)	6.6%	1.000 (Ref)	–
45-<60	310 (28.6%)	42 (42.0%)	352 (29.7%)	11.9%	1.916 (1.209, 3.038)	0.006
≥60	236 (21.8%)	20 (20.0%)	256 (21.6%)	7.8%	1.161 (0.662, 2.038)	0.602
Residence						
Urban	766 (70.7%)	68 (68.0%)	834 (70.4%)	8.2%	1.000 (Ref)	–
Rural	318 (29.3%)	32 (32.0%)	350 (29.6%)	9.1%	1.134 (0.730, 1.760)	0.577
Ethnicity						
Han	1069 (98.6%)	98 (98.0%)	1167(98.6%)	8.4%	1.000 (Ref)	–
Others	15 (1.4%)	2 (2.0%)	17 (1.4%)	11.8%	1.454 (0.328, 6.453)	0.622
Occupation						
Farmer	128 (11.8%)	10 (10%)	138 (11.7%)	7.2%	1.000 (Ref)	–
Enterprise employee	436 (40.2%)	38 (38%)	474 (40.0%)	8.0%	1.116 (0.561, 2.423)	0.767
Commercial service personnel	101 (9.3%)	9 (9.0%)	110 (9.3%)	8.2%	1.141 (0.438, 2.933)	0.783
Retiree	93 (8.6%)	9 (9.0%)	102(8.6%)	8.8%	1.239 (0.475, 3.191)	0.655
Unemployed/laid-off	101 (9.3%)	9 (9.0%)	110 (9.3%)	8.2%	1.141 (0.438, 2.933)	0.783
Others	225 (17.6%)	25 (25.0%)	250 (21.1%)	10.0%	1.422 (0.681, 3.193)	0.367
Educational level						
Illiterate/semi-literate	31 (2.9%)	3 (3.0%)	34 (2.9%)	8.8%	1.000 (Ref)	–
Primary school	101 (9.3%)	6 (6.0%)	107 (9.0%)	5.6%	0.614 (0.145, 2.599)	0.508
Junior high school	304 (28.0%)	37 (37.0%)	341 (28.8%)	10.9%	1.258 (0.366, 4.317)	0.716
High/technical secondary school	253 (23.3%)	26 (26.0%)	279 (23.6%)	9.3%	1.062 (0.304, 3.714)	0.925
College and above	395 (36.4%)	28 (28.0%)	423 (35.7%)	6.6%	0.732 (0.211, 2.545)	0.624
BMI						
<18.5	65 (6.0%)	3 (3.0%)	68 (5.7%)	4.4%	1.000 (Ref)	–
18.5-<24	654 (60.3%)	62 (62.0%)	716 (60.5%)	8.7%	2.054 (0.627, 6.726)	0.234
≥24	365 (33.7%)	35 (35.0%)	400 (33.8%)	8.8%	2.078 (0.621, 6.954)	0.236
Alcohol						
No	752 (69.4%)	73 (73.0%)	825 (69.7%)	8.8%	1.000 (Ref)	–
Drinking	246 (22.7%)	20 (20.0%)	266 (22.5%)	7.5%	1.001 (0.409, 2.45)	0.998
Quit drinking	86 (7.9%)	7 (7.0%)	93 (7.9%)	7.5%	1.194 (0.713, 1.999)	0.500
Smoking status						
No	698 (64.4%)	69 (69.0%)	767 (64.8%)	9.0%	1.000 (Ref)	
Smoking (daily)	261 (24.1%)	21 (21.0%)	282 (23.8%)	7.4%	1.243 (0.508, 3.042)	0.634
Smoking (non-daily)	70 (6.5%)	7 (7.0%)	77 (6.5%)	9.1%	0.678 (0.195, 2.352)	0.540
Quit smoking	55 (5.1%)	3 (3.0%)	58 (4.9%)	5.2%	1.229 (0.739, 2.043)	0.428
TB contact history						
No	900 (83.0%)	79 (79.0%)	979 (82.7%)	8.1%	1.000 (Ref)	–
Yes	37 (3.4%)	5 (5.0%)	42 (3.5%)	11.9%	0.65 (0.248, 1.699)	0.379
Unknown	147 (13.6%)	16 (16.0%)	163 (13.8%)	9.8%	0.805 (0.277, 2.341)	0.691
Underlying diseases						
No	869 (80.2%)	80 (80.0%)	949 (80.2%)	8.4%	1.000 (Ref)	–
Diabetes	51 (4.7%)	6 (6.0%)	57 (4.8%)	10.5%	1.278 (0.479, 2.852)	0.583
Diabetes, cardiovascular disease	9 (0.8%)	2 (2.0%)	11 (0.9%)	18.2%	2.414 (0.364, 9.566)	0.265
Diabetes, cardiovascular disease and kidney disease	1 (0.1%)	0 (0.0%)	1 (0.1%)	0.0%	0 (0, Inf)	–
Cardiovascular disease	122 (11.3%)	10 (10.0%)	132 (11.1%)	7.6%	0.890 (0.423, 1.687)	0.739
Kidney disease	3 (0.3%)	0 (0.0%)	3 (0.3%)	0.0%	0 (0, Inf)	–
Long-term immunosuppressant use	16 (1.5%)	0 (0.0%)	16 (1.4%)	0.0%	0 (0, Inf)	–
Other	13 (1.2%)	2 (2.0%)	15 (1.3%)	13.3%	1.671 (0.258, 6.189)	0.504
CD4/CD8 ratio						
<0.3	187 (17.3%)	9 (9.0%)	196 (16.5%)	4.6%	1.000 (Ref)	–
0.3 -<1	657 (60.6%)	54 (54.0%)	711 (60.1)	7.6%	1.708 (0.828, 3.523)	0.147
≥1	240 (22.1%)	37 (37.0%)	277 (23.4%)	13.4%	3.203 (1.508, 6.802)	0.002
CD8 cell count (cells/μL)						
M (P25,P75)	730 (518, 1038)	649 (436, 999)	718 (512, 1037)		1 (0.999, 1)	0.098
CD4 cell count (cells/μL)						
<200	133 (12.3%)	5 (5.0%)	138 (11.7%)	3.6%	1.000 (Ref)	–
≥200	951 (87.7%)	95 (95.0%)	1046 (88.3%)	9.1%	2.657 (1.062, 6.648)	0.037
HIV viral load (copies/mL)						
≤50	1000 (92.3%)	94 (94.0%)	1094 (92.4%)	8.6%	1.000 (Ref)	–
50-<1000	32 (3.0%)	4 (4.0%)	36 (3.0%)	11.1%	1.330 (0.460, 3.841)	0.598
≥1000	52 (4.8%)	2 (2.0%)	54 (4.6%)	3.7%	0.409 (0.098, 1.705)	0.220

### Immune status and HIV viral load of PLWHA

The median CD4*^+^* cell count in PLWHA was 460 cells/μL (IQR: 298–620 cells/μL), the median CD8*^+^* cell count was 718 cells/μL (IQR: 512–1037 cells/μL), and the median CD4/CD8 ratio was 0.63 (IQR: 0.38-0.97). The majority of PLWHA (88.3%, 1046/1184) were in the non-AIDS stage, but most PLWHA remained in a state of CD4/CD8 ratio inversion, CD4/CD8 ratio <1 (76.6%, 907/1184). The median HIV viral load was 50 copies/mL (IQR: 0–50 copies/mL), with most PLWHA in a state of HIV viral suppression (92.4%, 1094/1184) (**Table 1**).

### LTBI rate among PLWHA

Among 1184 PLWHA, the LTBI positivity rate was 8.4% (100/1184). The LTBI positivity rate in the <45 years age group was 6.6% (38/576), in the 45-<60 years age group was 11.9% (42/352), and in the ≥60 years age group was 7.81% (20/256). Univariate analysis revealed statistically significant differences in LTBI across different age groups (OR = 1.916, 95%*CI*: 1.209, 3.038, *P* = 0.006), CD4/CD8 ratios (OR = 3.203, 95%*CI*: 1.508, 6.802, *P* = 0.002), and CD4*^+^* cell count (OR = 2.657, 95%*CI*: 1.062, 6.648, *P* = 0.037). However, no statistically significant differences were found in LTBI regarding gender, residence, ethnicity, occupation, educational level, BMI, alcohol consumption, smoking status, TB contact history, underlying disease status, CD8*^+^* cell count, or HIV viral load (**Table 1**).

### Multivariate analysis of influencing factors for LTBI in PLWHA

**Figure 1:**
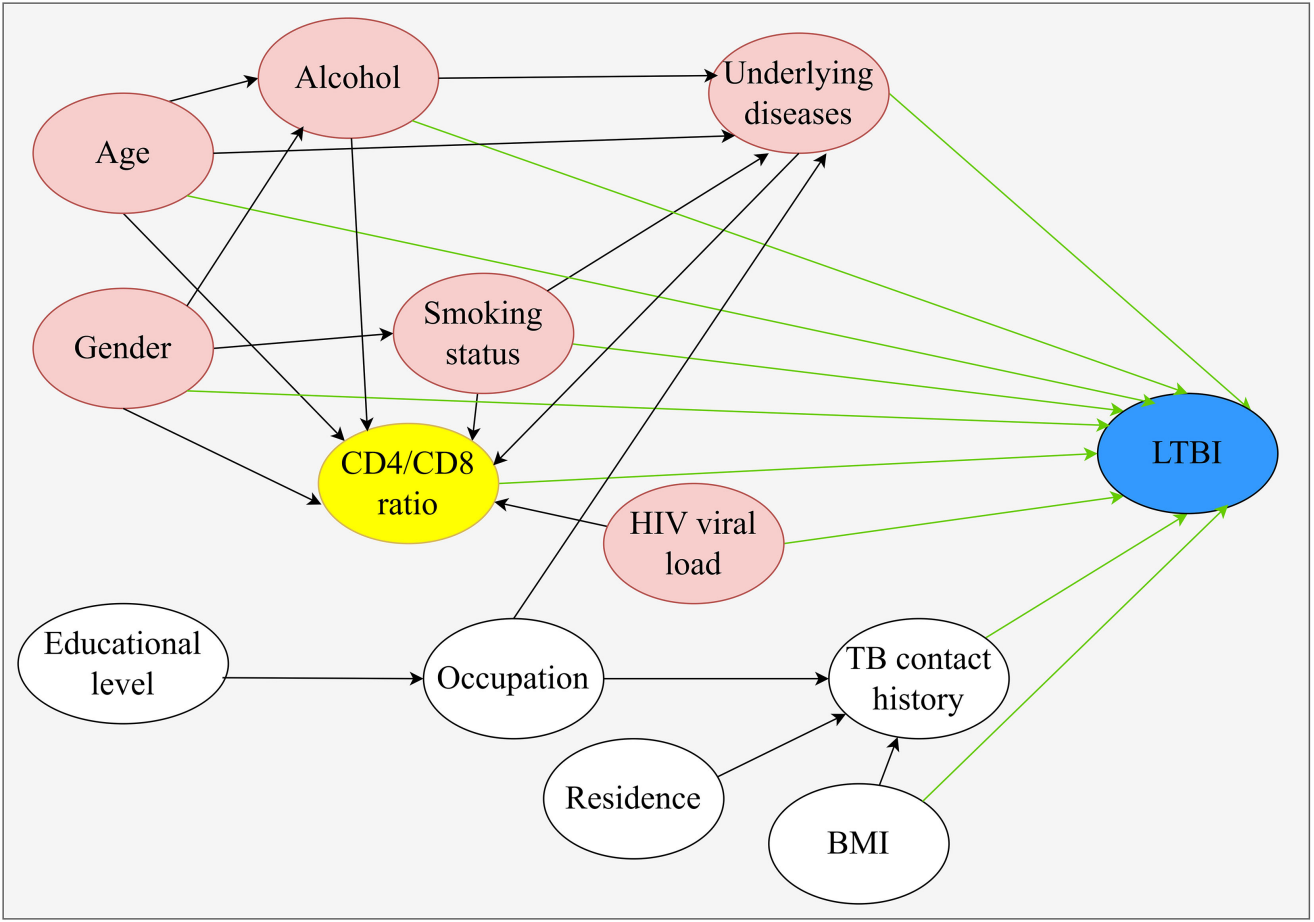
Directed acyclic graph (DAG) illustrating the relationship between CD4/CD8 ratio and LTBI risk.

To achieve a more comprehensive and accurate assessment of the independent effects of individual variables on LTBI risk, we use DAG, constructed based on causal knowledge from previous studies (Moran et al., 2022; Vassallo et al., 2025), to guide the inclusion of variables in the multivariate logistic regression model. Since the CD4/CD8 ratio is directly calculated from the CD4 and CD8 cell counts, to avoid overadjustment, we did not include the CD4 and CD8 cell counts as variables in the DAG. Additionally, evidence regarding the association between ethnicity and the CD4/CD8 ratio or LTBI in PLWHA is limited, so we did not include ethnicity in the DAG. In the final DAG, the yellow nodes represent the exposure factors being studied, the blue nodes represent the outcome variables of this study, the red nodes indicate confounding factors in the causal chain, and the green arrows represent the associations with the outcome. Based on this DAG, the identified confounders were age, gender, BMI, smoking status, alcohol consumption, underlying diseases, and HIV viral load. Furthermore, no mediator variables were identified in these variables. These confounders were then included in the multivariate analysis to explore the relationship between the CD4/CD8 ratio and LTBI risk (**Figure 1**). Multivariate analysis was performed with LTBI as the dependent variable (negative = 0, positive = 1). The results showed that age 45-<60 years (OR = 2.158, 95% *CI*: 1.339-3.478, *P* = 0.002) and CD4/CD8 ratio ≥1 (OR = 3.562, 95% *CI*: 1.627-7.800, *P* = 0.001) may be independent influencing factors for LTBI (**Table 2**).

**Table 2:** Multivariate logistic regression analysis of risk factors associated with LTBI among PLWHA.

Variable	OR	95%*CI*	*Z*	*P*
Gender				
Male	Ref	–	–	–
Female	0.780	(0.372, 1.632)	-0.661	0.509
Age				
<45	Ref	–	–	–
45-<60	2.158	(1.339, 3.478)	3.160	0.002
≥60	1.264	(0.68, 2.35)	0.741	0.459
Alcohol				
No	Ref	–	–	–
Drinking	1.024	(0.400, 2.617)	0.049	0.961
Quit drinking	1.128	(0.649, 1.959)	0.427	0.669
Smoking status				
No	Ref	–	–	–
Smoking (daily)	1.342	(0.535, 3.367)	0.628	0.530
Smoking (non-daily)	0.677	(0.185, 2.481)	-0.588	0.556
Quit smoking	1.371	(0.789, 2.38)	1.119	0.263
Underlying diseases				
No	Ref	–	–	–
Diabetes	1.228	(0.492, 3.065)	0.440	0.660
Diabetes, cardiovascular disease	3.052	(0.597, 15.606)	1.340	0.180
Diabetes, cardiovascular disease and kidney disease	0	(0, Inf)	-0.006	0.995
Cardiovascular disease	0.808	(0.397, 1.643)	-0.590	0.555
Kidney disease	0	(0, Inf)	-0.011	0.992
Long-term immunosuppressant use	0	(0, Inf)	-0.024	0.981
Other	2.094	(0.444, 9.867)	0.934	0.350
CD4/CD8 ratio				
<0.3	Ref	–	–	–
0.3 -<1	1.780	(0.846, 3.747)	1.519	0.129
≥1	3.562	(1.627, 7.800)	3.177	0.001
HIV viral load(copies/mL)				
<50	Ref	–	–	–
50-<1000	1.908	(0.635, 5.731)	1.151	0.250
≥1000	0.579	(0.135, 2.491)	-0.734	0.463

### Dose-response relationships between CD4^+^ and CD8^+^ cell counts and LTBI risk

**Figure 2:**
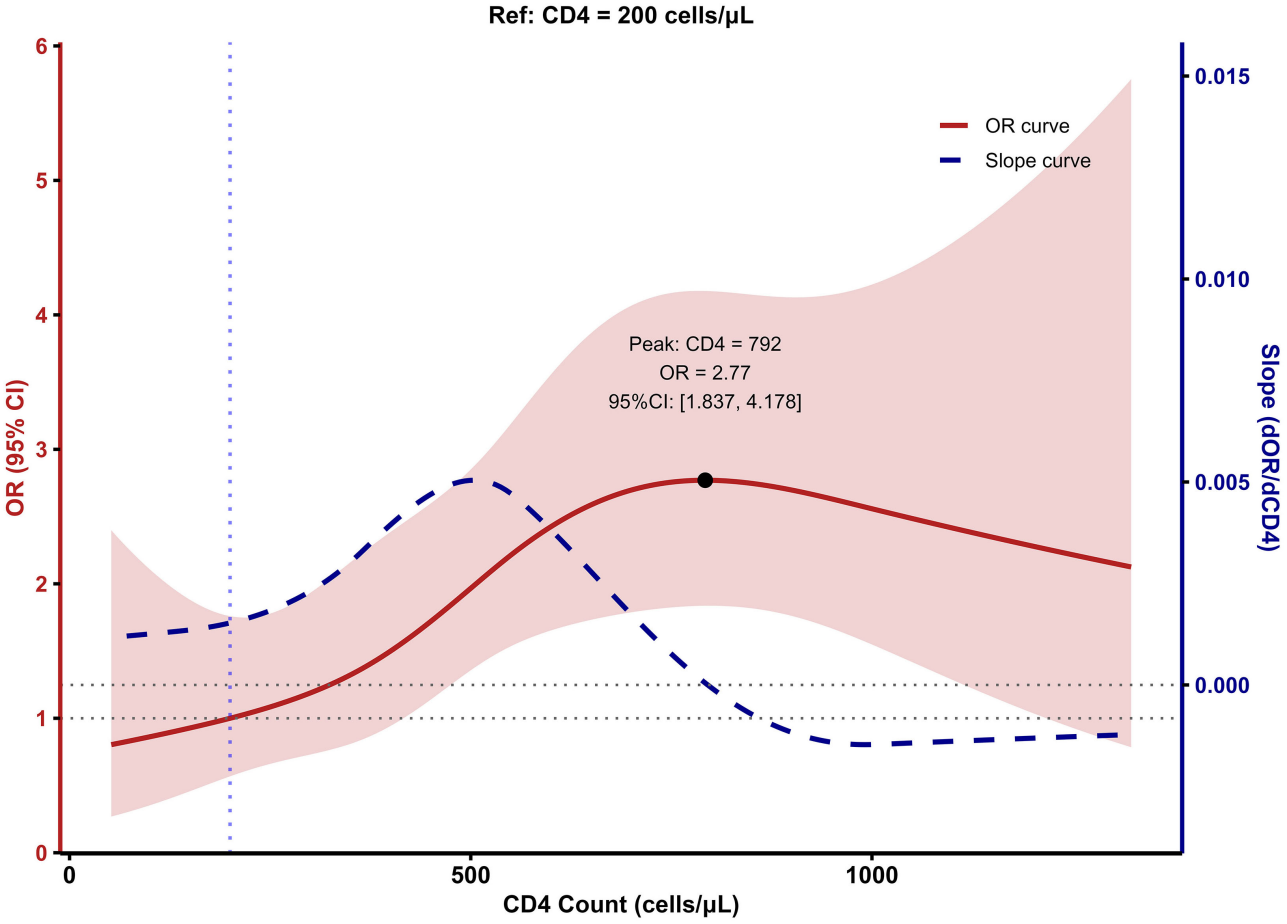
Relationship between CD4 cell Count and LTBI risk based on RCS model.

**Figure 3:**
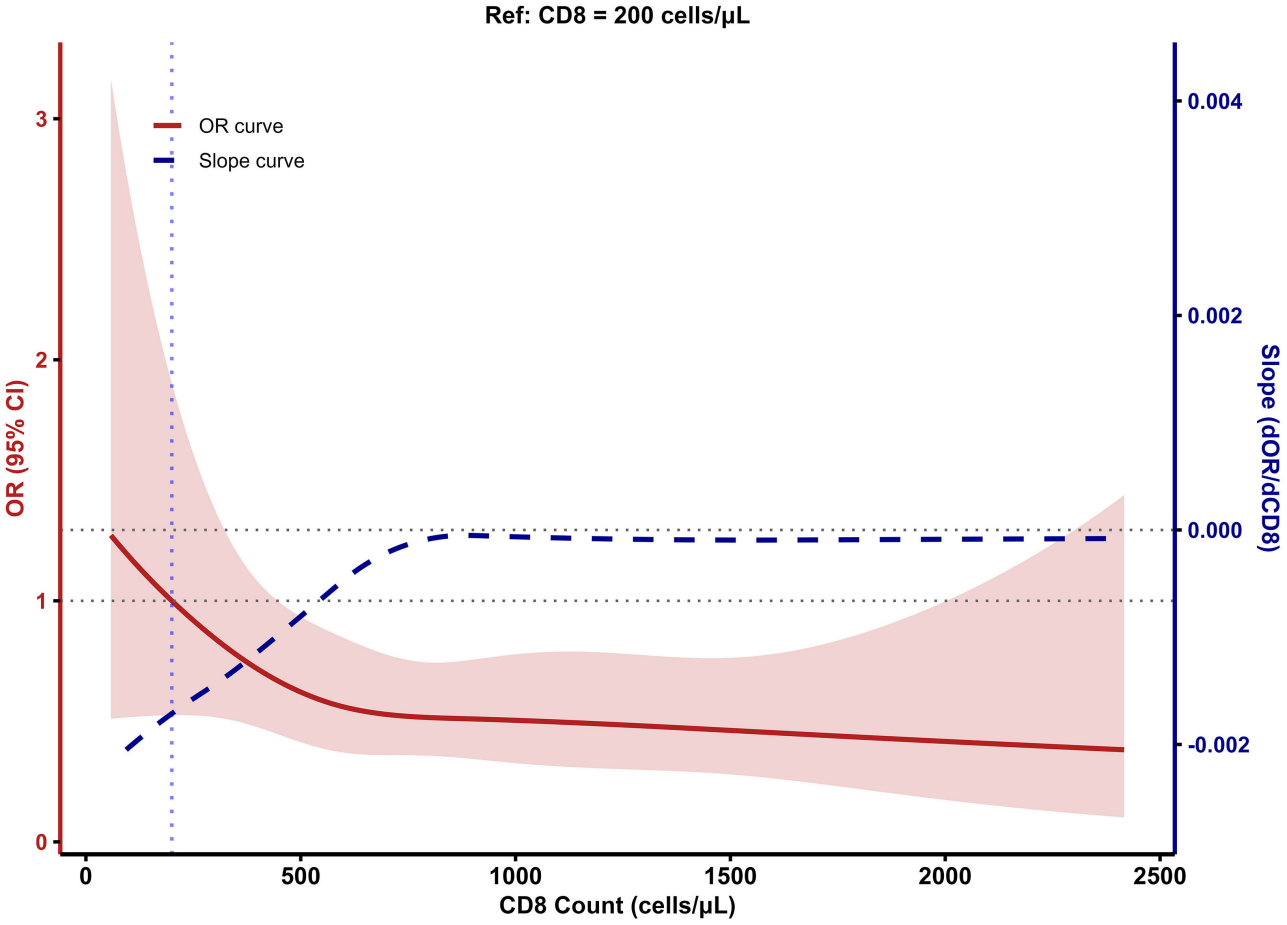
Relationship between CD8 cell Count and LTBI risk based on RCS model.

To evaluate the dose–response relationships between CD4^+^ and CD8^+^ T-cell counts and LTBI risk, we fitted restricted cubic spline (RCS) models with CD4^+^ and CD8^+^ counts as continuous predictors and plotted both the odds ratio (OR) curves and their first derivatives (slope curves) on dual y-axes (**Figures 2**, **3**). In these models, the ORs varied nonlinearly across the ranges of CD4^+^ and CD8^+^ counts. The confidence intervals were relatively wide at the extremes, due to the smaller number of participants in these ranges; therefore, estimates at the boundaries should be interpreted with caution.

The RCS for CD4^+^ T cells revealed a nonlinear association between CD4^+^ cell count and LTBI risk in PLWHA. Across all PLWHA, the risk of LTBI increased with rising CD4^+^ cell count, peaking at 792 cells/μL (OR = 2.77), after which LTBI risk gradually declined (**Figure 2**). The corresponding slope curve was positive at lower CD4^+^ counts, crossed zero near the peak, and became negative at higher counts, confirming this rise-and-fall pattern. For CD8^+^ T cells, the dose–response curve showed a decreasing trend in LTBI risk with increasing CD8^+^ cell count, which plateaued when the CD8^+^ count reached around 500 cells/μL (**Figure 3**). Consistently, the slope curve was strongly negative at low CD8^+^ counts and approached zero beyond approximately 500 cells/μL, indicating little additional change in LTBI risk at higher CD8^+^ levels.

### Dose-response relationship between CD4/CD8 ratio and LTBI risk

**Figure 4:**
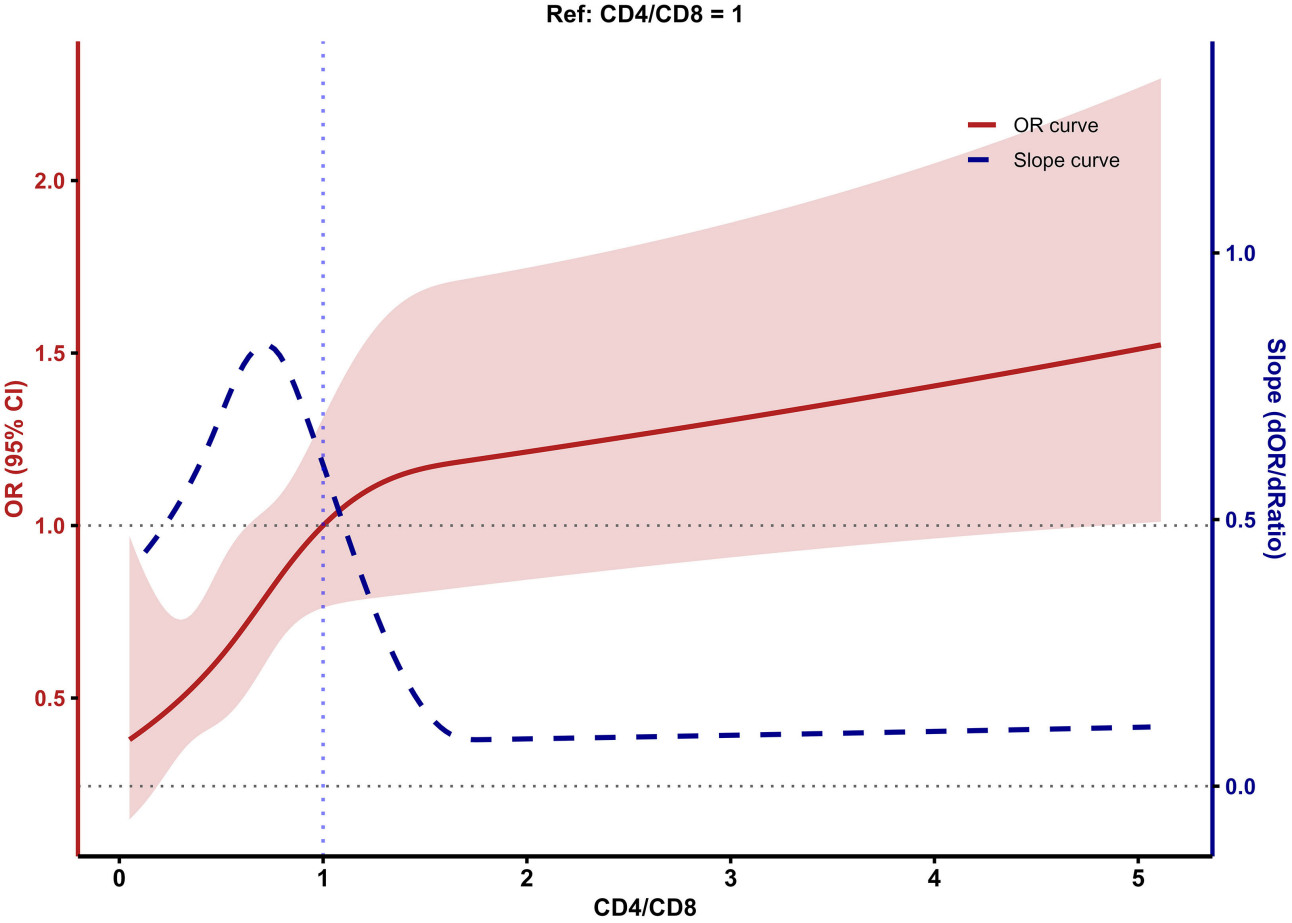
Relationship between CD4/CD8 ratio and LTBI risk based on RCS model.

Using a similar RCS approach, we examined the dose-response relationship between the CD4/CD8 ratio and LTBI risk and again plotted both the OR and slope curves (**Figure 4**). LTBI risk exhibited a nonlinear increasing trend with rising CD4/CD8 ratio. When the CD4/CD8 ratio was <1, the OR curve and a clearly positive slope indicated a pronounced increase in LTBI risk as the ratio increased. When the CD4/CD8 ratio was ≥1, the slope curve approached zero, and the OR curve rose more gradually, suggesting that the increase in LTBI risk slowed at higher ratios. As with CD4^+^ and CD8^+^ counts, the confidence intervals widened at the extremes of the CD4/CD8 ratio distribution, so estimates in these ranges should be interpreted cautiously.

## Discussion

Through this cross-sectional study conducted among PLWHA, we found that PLWHA aged 45-<60 years had significantly higher LTBI risk compared to those <45 years old. Notably, the dose-response relationship between CD4^+^ cell count and LTBI risk showed an increasing trend, while the dose-response relationship between CD8^+^ cell count and LTBI risk demonstrated a decreasing trend. Furthermore, a significant non-linear dose-response relationship was observed between CD4/CD8 ratio and LTBI risk, with LTBI risk in PLWHA showing an upward trend as the CD4/CD8 ratio increased.

Among the 1184 PLWHA included in this study, the LTBI rate detected by QFT was 8.4%, which was lower than the LTBI rates of PLWHA reported in Ningxia (24.4%) (Jiang et al., 2022), Changzhou (10.02%) (Zhang et al., 2020), and Xian (8.8%) (Xin et al., 2017) in China. Jiangsu Province is a low TB prevalence area (Xiong et al., 2024), which may be one of the reasons for the relatively low LTBI rate among PLWHA in this survey.

Multivariable analysis showed that, compared with PLWHA aged <45 years, those aged 45 to <60 years had higher risk of LTBI (OR = 2.158; 95% CI: 1.339-3.478; P = 0.002). This corresponds to approximately a two-fold increase in the odds of LTBI among PLWHA aged 45-<60 years. The 95% confidence interval is relatively narrow, indicating reasonable precision of the estimate. This finding is consistent with related research findings (Mancuso et al., 2016).In China, the first national TB epidemiological survey, conducted in 1979, reported an active pulmonary TB prevalence of 717 per 100,000, indicating widespread community transmission at that time. Subsequent national surveys in 1984/1985, 1990, 2000, and 2010 showed only gradual declines in TB prevalence (Tu, 2013). Taken together, these findings suggest that TB prevalence and community transmission in the 1960s and 1970s were higher than in later decades, and that adults currently aged 45 to 60 years, most of whom were born in the 1960s and 1970s, may therefore have faced a higher risk of TB exposure earlier in life and accumulated latent infection risk. Meanwhile, the body experiences accumulated TB exposure risk with age and age-related subtle changes in the immune system [such as immunosenescence (Fulop et al., 2018) or inflammaging (Li et al., 2023)]. Research has shown that increasing age is a major risk factor for developing and dying from TB, as aging is accompanied by declining immune system function, making elderly individuals more susceptible to TB infection (Li et al., 2019). Furthermore, middle-aged PLWHA show significantly slower CD4*^+^* T cell recovery after antiretroviral therapy compared to younger PLWHA (Manfredi, 2002), suggesting that even with antiretroviral treatment, their immune reconstitution remains relatively insufficient, potentially making latent TB difficult for the body to clear or control.

Studies have found that the CD4/CD8 ratio is increasingly recognized as a more sensitive and comprehensive indicator of immune health than CD4^+^ cell count (Luo et al., 2024) and can serve as a biomarker for non-AIDS-related diseases (Mussini et al., 2015). In the present study, we observed that multivariate analysis showed that PLWHA with CD4/CD8 ratio ≥1 had markedly higher odds of LTBI than those with CD4/CD8 ratio <0.3 (OR = 3.562; 95% CI: 1.627-7.800), indicating more than a three-fold increase in odds. However, the 95% confidence interval is relatively wide, suggesting considerable uncertainty around the magnitude of the association. Therefore, this estimate should be interpreted cautiously and verified in larger studies. This finding is consistent with previous studies’ finding that immune recovery is independently associated with QFT positivity rate (Danel et al., 2014; Mthembu et al., 2023). The mechanism may be related to enhanced CD4*^+^* T cell-mediated immune responses. The core principle of IGRAs is to detect the ability of T cells to produce IFN-γ after stimulation with *Mycobacterium tuberculosis*-specific antigens (Chinese Society for Tuberculosis, Chinese Medical Association, 2022). When the CD4/CD8 ratio ≥1, CD4*^+^* T cell-dominated Th1-type immune responses can promote interferon-gamma release after stimulation with TB-specific antigens (ESAT-6/CFP-10) (Prezzemolo et al., 2014), thereby improving QFT detection sensitivity. Conversely, a CD4/CD8 ratio <1 suggests that excessive activation of CD8*^+^* T lymphocytes may lead to immune exhaustion, suppress antigen-specific responses, and increase false-negative risk (Serrano-Villar et al., 2014). A CD4/CD8 ratio ≥1 in this study may more likely indicate a detectable IFN-γ sensitization state rather than increased LTBI incidence risk. A study by Lu P et al. indirectly supports this view (Lu et al., 2025), demonstrating that QFT detection sensitivity remains relatively stable in PLWHA with relatively high CD4*^+^* cell count. This suggests that a higher CD4/CD8 ratio may reflect sufficient immune response capacity to support accurate QFT detection results. A low CD4/CD8 ratio in PLWHA may signal higher true TB risk (Liu et al., 2023), but it can also impair test sensitivity, meaning both clinicians and researchers must carefully factor immune status into LTBI screening. Considering epidemiologic risk and immune function is needed to avoid mistaking immune-related test variations for differences in actual TB infection. This study further analyzed differences in QFT detection results at different CD4/CD8 ratio levels and found no statistically significant difference in QFT positivity rates between PLWHA with CD4/CD8 ratio <0.3 and those with 0.3≤CD4/CD8 ratio <1. This result suggests that in immune imbalance states where the CD4/CD8 ratio is below 1, the body’s immune response to TB antigens is uniformly significantly limited, with consistently elevated false-negative risk in IGRA detection. Therefore, individuals with CD4/CD8 ratio <0.3 and 0.3≤CD4/CD8 ratio <1 can be considered to have the same immune imbalance level. CD4/CD8 ratio ≥1 may be the optimal threshold for screening LTBI risk in PLWHA, while immune imbalance below this value may lead to increased false-negative risk in QFT detection.

To further explore the relationship between CD4/CD8 ratio and LTBI risk in PLWHA, we used a restricted cubic spline (RCS) model to fit the dose-response relationship between CD4^+^ cell count, CD8^+^ cell count, CD4/CD8 ratio, and LTBI risk. The results showed a non-linear relationship between CD4^+^ cell count and LTBI risk. As CD4^+^ cell count increases, the LTBI risk in PLWHA increases, peaking when CD4^+^ cell count reaches 793 cells/μL, and then the risk decreases. Jiang et al. (2022) demonstrated that within a certain range, when CD4^+^ cell count is lower, the risk of LTBI in PLWHA gradually increases as CD4^+^ cell count rises, which is consistent with the trend observed in this study. Additionally, compared with PLWHA whose CD4^+^ cell count <800cells/μL, LTBI risk begins to decrease after the CD4^+^ cell count reaches 800 cells/μL in PLWHA, which is similar to the finding in this study (Jiang et al., 2022). This may be because PLWHA have impaired immune function, with suppressed cellular immunity and hypersensitivity reactions, leading to a weak or even absent QFT response and thus a lower LTBI risk. As the CD4^+^ cell count increases, the sensitivity of QFT diagnosis also improves (Song, 2025). As CD4^+^ cell count recover with ART, the risk of tuberculosis infection gradually decreases, and QFT positive rates decline, indicating a reduced risk of LTBI (Suthar et al., 2012).

The CD4/CD8 ratio in PLWHA showed a significant non-linear dose-response relationship with LTBI risk. As the CD4/CD8 ratio increases, the risk of LTBI increases. Wong et al. (2019) found that increased LTBI incidence correlates with higher CD4/CD8 ratios, consistent with our findings. The reason for this is similar to the previous explanation: due to immune impairment in PLWHA, cellular immunity and hypersensitivity reactions are suppressed, leading to a weak or absent QFT response and lower positivity rates, as confirmed by Yu et al. (2022). In the RCS model, participants with CD4/CD8 ratios < 1 showed lower odds of QFT positivity compared with those with higher ratios. In the context of PLWHA, CD4/CD8 ratio < 1 generally reflects incomplete immune reconstitution and more pronounced immune dysfunction, which can reduce the sensitivity of the QFT assay and increase the likelihood of false-negative results (Castilho et al., 2022). Thus, the lower odds of QFT positivity in individuals with CD4/CD8 < 1 likely reflect reduced ability to mount a detectable IFN-γ response, rather than a lower underlying risk of LTBI. as the CD4/CD8 ratio increases, immune recovery progresses, enhancing diagnostic sensitivity (Yu et al., 2022). The RCS model showed that when the CD4/CD8 ratio ≥1, the increase in LTBI risk gradually slows down, reinforcing the idea that CD4/CD8 ≥1 could be the threshold for LTBI screening in PLWHA. In the RCS results, the confidence intervals around the spline curves were relatively wide at the lower and higher ends of CD4, CD8, and CD4/CD8. The smaller number of participants in these extreme ranges results in reduced precision of the estimates. Consequently, the apparent non-linear trends at the tails of the curves should be viewed as exploratory, and future studies with larger sample sizes in these ranges are needed to confirm these findings.

This study has several limitations. First, the cross-sectional design only provides a snapshot of CD4*^+^* and CD8*^+^* cell counts, viral load, and QFT results at one point in time. Therefore, it is not possible to determine temporal relationships or draw causal inferences between immune status, viral replication, and QFT positivity. Additionally, the Chinese Center for Disease Control and Prevention reports that the male-to-female ratio among newly reported HIV infection cases in 2024 was 3.2:1 (approximately 23.8% female) (National Center for AIDS/STD Control and Prevention, Chinese Center for Disease Control and Prevention, 2025). However, in our study, the proportion of females recruited was only 9.9%, which is not representative of the general population. This gender imbalance limits the statistical power for sex-stratified analyses and may reduce the generalizability of our findings to females.

In conclusion, this cross-sectional study explored factors associated with QFT-defined LTBI risk in PLWHA and applied a restricted cubic spline model to characterize potential non-linear relationships. We observed that PLWHA aged 45-<60 years appeared to have a relatively higher risk of QFT-defined LTBI, and that a CD4/CD8 ratio≥1 might represent a more favorable range for identifying LTBI risk in this population. If confirmed in future prospective studies, these findings may support a more targeted approach when performing LTBI screening using QFT in PLWHA, with particular attention to middle-aged PLWHA (45-<60 years) and consideration of stratified screening strategies: (1) In PLWHA with a CD4/CD8 ratio ≥1, QFT detection alone can be used; (2) In those with a CD4/CD8 ratio <1, it may be prudent to combine QFT with other TB-specific tests and to monitor changes in the CD4/CD8 ratio over time. Given the cross-sectional design and the use of QFT positivity as an operational definition of LTBI, these recommendations should be viewed as exploratory, and future studies incorporating multidimensional immune markers and clinical outcomes are needed to further validate their application value.

## Data Availability

The data that support the findings of this study are available on request from the corresponding author. The data are not publicly available due to privacy or ethical restrictions. Requests to access these datasets should be directed to ZX, Xzp15951589696@163.com.
